# Postcardiac injury syndrome caused by radiofrequency catheter ablation of persistent atrial fibrillation: severe pulmonary arterial hypertension with severe tricuspid regurgitation: a rare case report and literature review

**DOI:** 10.1186/s12872-023-03202-1

**Published:** 2023-04-13

**Authors:** Jia Shi, Mengjiao Shao, Xianhui Zhou, Yanmei Lu, Baopeng Tang

**Affiliations:** 1 Cardiac Pacing and Physiological Department, The First Affiliated Hospital of Xinjiang Medical University, No.137, South Liyushan Road, Xinshi Zone, Urumqi, Xinjiang China; 2 Xinjiang Key Laboratory of Cardiac Electrophysiology and Cardiac Remodeling, The First Affiliated Hospital of Xinjiang Medical University, Urumqi, Xinjiang China; 3 Department of Cardiology, The Fifth Affiliated Hospital of Sun Yat-sen University, Zhuhai, China; 4 State Key Laboratory of Pathogenesis, Prevention and Treatment of High Incidence Diseases in Central Asia, Xinjiang Medical University, Urumqi, Xinjiang China

**Keywords:** A case report, Literature review, Postcardiac injury syndrome, Radiofrequency catheter ablation, Persistent atrial fibrillation

## Abstract

**Background:**

Postcardiac injury syndrome (PCIS) is an easy-to-miss diagnosis, but it is not an uncommon complication. The phenomenon of echocardiography (ECHO) showing both severe pulmonary arterial hypertension (PAH) and severe tricuspid regurgitation (TR) is indeed rare in PCIS after extensive radiofrequency ablation.

**Case presentation:**

A 70-year-old male was diagnosed with persistent atrial fibrillation. The patient received radiofrequency catheter ablation due to his atrial fibrillation being refractory to antiarrhythmic drugs. After the anatomical three-dimensional models were created, ablations were performed on the left and right pulmonary veins, roof linear and bottom linear of the left atrium, and the cavo-tricuspid isthmus. The patient was discharged in sinus rhythm (SR). After 3 days, he was admitted to the hospital for gradually worsening dyspnea. Laboratory examination showed a normal leukocyte count with an increased percentage of neutrophils. The erythrocyte sedimentation rate, C-reactive protein concentration, interleukin-6, and N-terminal pro-B-type natriuretic peptide were elevated. ECG exhibited SR, V_1_-V_4_ of precordial lead P-wave amplitude which was increased but not prolonged, PR segment depression, and ST-segment elevation. Computed tomography angiography of the pulmonary artery revealed that the lung had scattered high-density flocculent flakes and a small amount of pleural and pericardial effusion. Local pericardial thickening was seen. ECHO showed severe PAH with severe TR. Diuretics and vasodilators did not relieve the symptoms. Tumors, tuberculosis, and immune system diseases were all excluded. Considering the patient’s diagnosis of PCIS, the patient was treated with steroids. The patient recovered on the 19th day post ablation. The patient’s condition was maintained until 2 years of follow-up.

**Conclusions:**

The phenomenon of ECHO showing severe PAH with severe TR is indeed rare in PCIS. Due to the lack of diagnostic criteria, such patients are easily misdiagnosed, leading to a poor prognosis.

**Supplementary Information:**

The online version contains supplementary material available at 10.1186/s12872-023-03202-1.

## Background

Postcardiac injury syndrome (PCIS) is an immune-mediated inflammatory response syndrome in the pericardium, pleura, and/or lung parenchyma. The underlying mechanisms of PCIS remain unclear. The incidence of PCIS may be associated with the extent of myocardial injury and may be higher in procedures that involve larger areas of the myocardium, and these procedures include pericardiotomy and cardiac interventional therapy, such as catheter ablation [1]. None of the previous studies reported severe pulmonary arterial hypertension (PAH) accompanied by severe tricuspid regurgitation (TR) as the primary sign of PCIS after radiofrequency catheter ablation (RFCA) for persistent atrial fibrillation (persAF), as in this case.

## Case presentation

**Fig. 1:**
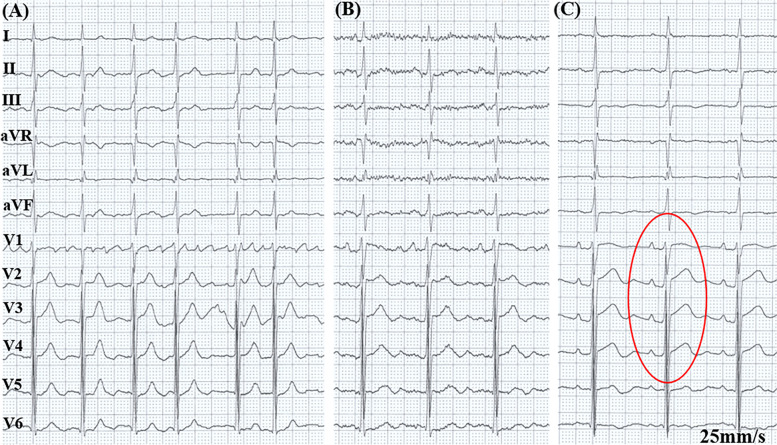
**A** Admission surface ECG showed atrial fibrillation. **B** Postoperative ECG showed SR. **C** V1-V4 of the precordial lead showed the P-wave amplitude which was increased but not prolonged, PR segment depression, and ST-segment elevation when the patient was hospitalized again (red oval). ECG, Electrocardiogram; SR, Sinus rhythm

**Fig. 2:**
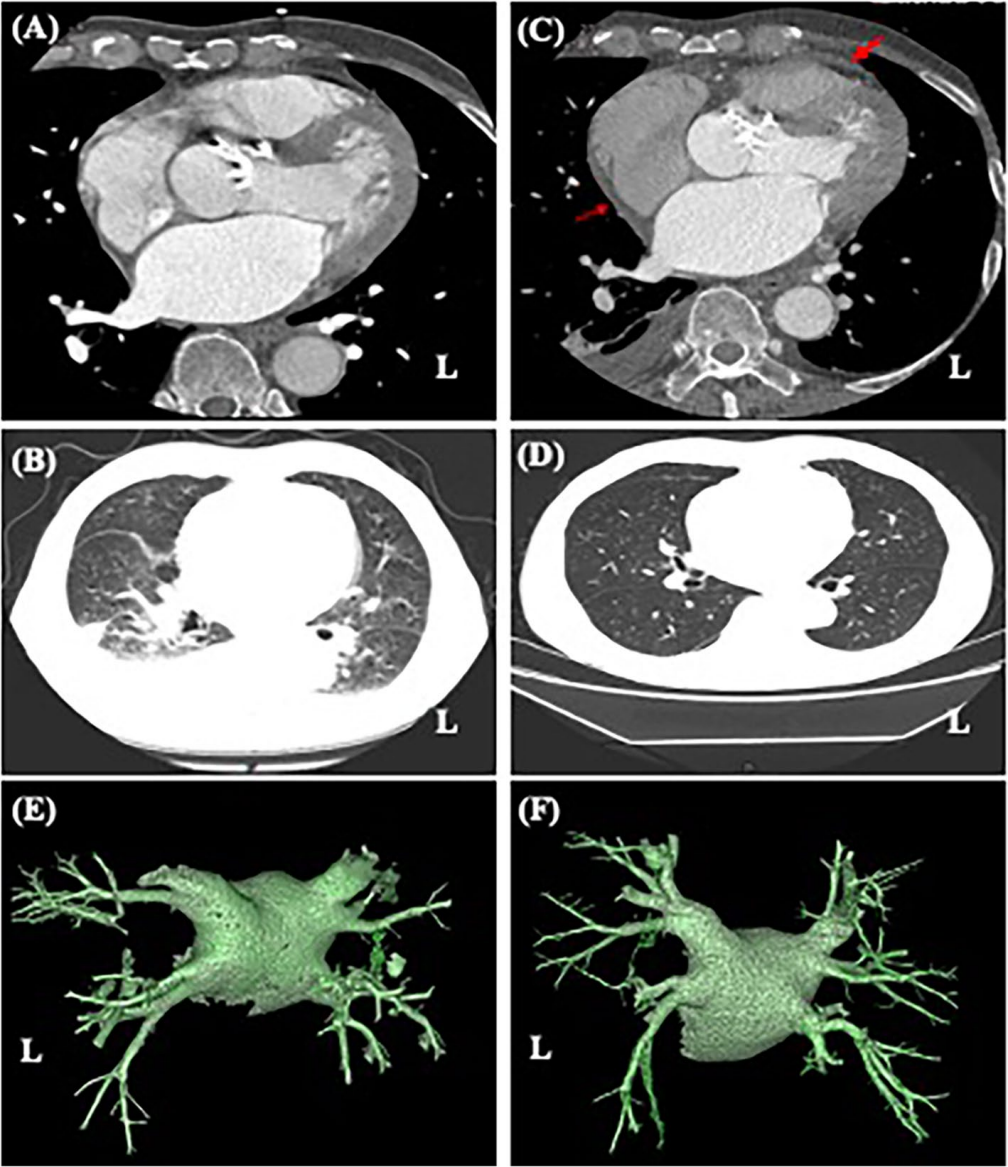
Representative images from the CT of the chest before pulmonary vein isolation, during PCIS, and after relief. **A** Preoperation: CT angiography of the pulmonary vein showed that the pericardial and bilateral lung fields were normal. During PCIS: **B** local pericardial thickening (red arrows) and bilateral pleural effusion were seen. **C** CT angiography of the pulmonary vein showed bilateral patchy areas of high-density flocculent flakes, which were distributed mainly in the inferior lobe of the right lung. **D** Improvement: A chest CT scan performed 19 days later showed that the lesions of the lung parenchyma had subsided. **E** Preoperation: 3D image of left atrium and pulmonary veins. **F** During PCIS: 3D image of left atrium and pulmonary veins. CT, Computed tomography; PCIS, Postcardiac injury syndrome

**Fig. 3:**
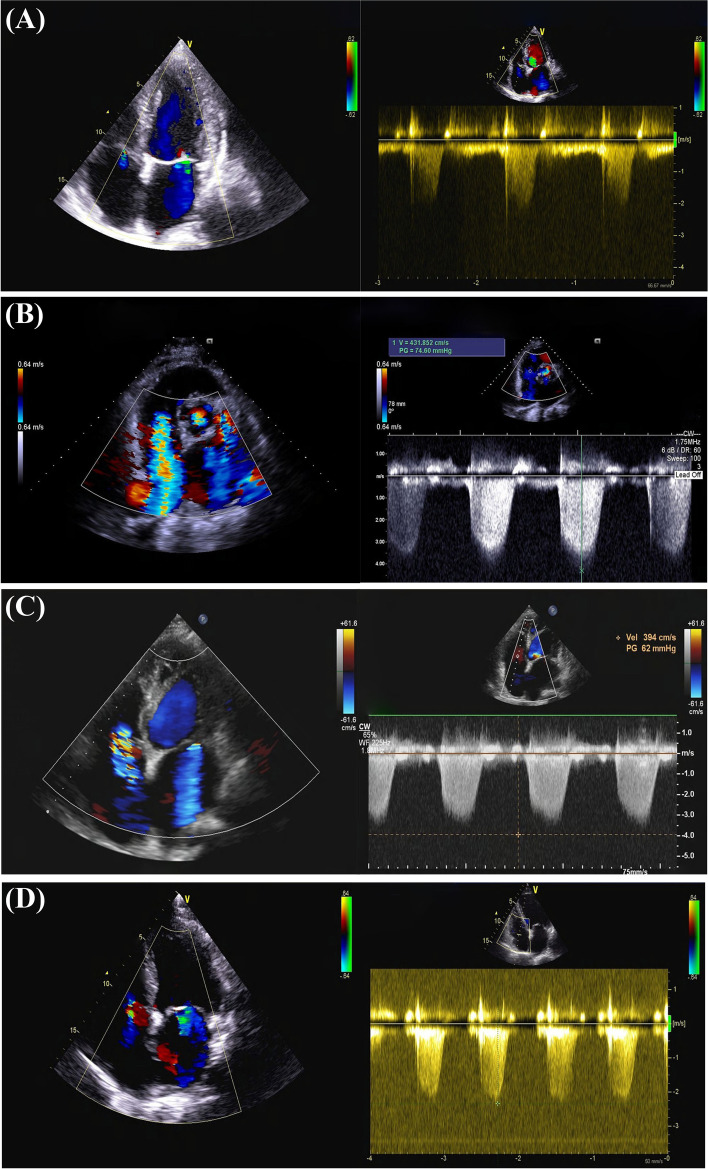
Representative images from echocardiography before pulmonary vein isolation and during PCIS. Preoperation: **A** Mild regurgitation of the tricuspid valve with normal PAH. During PCIS: **B** Severe TR (maximum regurgitation pressure difference 75 mmHg). Severe PAH (estimated 85 mmHg). **C** The TR (maximum regurgitation pressure difference 62 mmHg) and PAH (estimated 72 mmHg) were alleviated. **D** Mild TR (maximum regurgitation pressure difference 22 mmHg). PAH recovered. PCIS, Postcardiac injury syndrome. TR, Tricuspid regurgitation. PAH, Pulmonary arterial hypertension

A 70-year-old male diagnosed with persAF with a 3-month history of symptomatic palpitations received RFCA because his arrhythmia was refractory to antiarrhythmic drugs (Fig. 1A). He had been receiving anticoagulants (Rivaroxaban, Xarelto, 20 mg once a day, oral) regularly for 3 weeks. There were no additional comorbidities or relevant family history in this patient. Computed tomography angiography (CTA) of the pulmonary vein was normal (Fig. 2A, E). Echocardiography (ECHO) revealed an ejection fraction of 56%, a left atrium (LA) anteroposterior diameter of 40 mm, a calcified aortic valve, and a mild insufficiency of the mitral and tricuspid valves (Fig. 3A).

A three-dimensional anatomical of the LA and the pulmonary veins (PVs) was created by the CARTO® 3 System (Biosense Webster, Irvine, CA, USA). Ablations were performed on the left and right PVs, roof linear and bottom linear of the LA. After the anatomical three-dimensional model of the right atrium and the tricuspid annulus were prepared, ablation was performed on the cavo-tricuspid isthmus (CTI). Electrophysiological mapping confirmed the circumferential pulmonary vein isolation and CTI line bidirectional block. The postablation electrocardiogram (ECG) showed sinus rhythm (SR) (Fig. 1B). The patient continued to take oral anticoagulants, and the patient was discharged 2 days later.

On the 5^th^ day postablation, he was admitted to the hospital after he complained of dyspnea and fatigue, which gradually worsened to paroxysmal nocturnal dyspnea, but he had no fever. His heart rate was below 110 beats per minute. Physical examination showed a pansystolic murmur that was heard in the fourth intercostal space at the left edge of the sternum and decreased breath sounds at the base of the lungs. Laboratory examination showed a normal leukocyte count and absolute number of neutrophils (5.78 × 10^9^/L) with an increased percentage of neutrophils (79.6%). The level of SO_2_ was normal. The levels of myocardial enzymes were negative, including hypersensitive troponin T and creatine kinase isoenzymes. The erythrocyte sedimentation rate (ESR, 18 mm/h, normal value 0–15 mm/h), C-reactive protein concentration (CRP, 13.8 mg/L, normal value 0–6 mg/L), interleukin-6 (IL-6, 126.6 pg/mL, normal value 0–7 pg/mL) and N-terminal pro-B-type natriuretic peptide (2200 ng/L, normal value 0-125 ng/L) were elevated. ECG exhibited SR, V_1_-V_4_ of precordial lead P-wave amplitude which was increased but not prolonged, PR segment depression, and ST-segment elevation (Fig. 1C). CTA of the pulmonary artery revealed no signs of embolism. No stenosis was observed on CTA of the PVs (Fig. 2F). The lung had scattered high-density flocculent flakes and a small amount of pleural effusion (Fig. 2B), and local pericardial thickening was seen (Fig. 2C). ECHO showed an ejection fraction of 53%, severe TR (maximum regurgitation pressure difference 75 mmHg), severe PAH (estimated 85 mmHg) (Fig. 3B), and mild pericardial effusion. Tuberculosis infectious T-lymphocyte spot assay and autoimmune test results were negative.

As a result of these examinations, tumors, tuberculosis, immune system diseases, acute coronary syndrome, pulmonary vein stenosis, and acute pulmonary embolism were excluded. The patient was initially diagnosed with acute heart failure with acute pulmonary edema and was treated with a diuretic and a vasodilator along with anticoagulants. However, the treatment did not relieve the symptoms, and the diagnoses of severe PAH and TR were difficult to explain. Thus, nonspecific inflammation within large areas of the atrial myocardium after RFCA could not be ruled out in this case. Hence, the patient was started on intravenous corticosteroid (Methylprednisolone, 80 mg per day) therapy, along with adjuvant therapy including a low dose of diuretics. The patient’s symptoms began to be relieved on the 7th day postablation, after steroids had been given for one day. On the 9th day postablation, the above symptoms were considerably alleviated, and all indexes gradually returned to normal. ECHO showed that the TR and PAH were alleviated (Fig. 3C). The patient’s steroid therapy was tapered slowly. On the 14th day postablation, the patient’s ECHO index recovered (Fig. 3D). The patient’s pleural effusion regressed completely on the 16th day postablation. Chest CT was repeated on the 19th day, which revealed that the original infiltration of the lung parenchyma had subsided (Fig. 2D). The oral steroid was maintained at the minimal effective dose for a week and then was withdrawn. The patient was followed up every 3 months and has been followed up for 2 years to date. He has resumed daily activities, and he has maintained a SR on his ECG.

## Discussion and conclusions

PCIS is an easy-to-miss diagnosis but not an uncommon complication, and it is characterized by fever, chest pain, pericardial effusion, and elevated inflammatory markers, as discussed in our review [1]. In contrast, we report a rare case of severe PAH accompanied by severe TR as the primary sign of PCIS after RFCA for persAF. In our review, we found that PCIS occurs commonly after cardiovascular implantable electronic device implantation, percutaneous coronary intervention, transcatheter valve replacement/repair, and catheter ablation in predisposed individuals [2–11]. With the increasing use of catheter intervention for the treatment of cardiovascular disease, it is more important than ever to be aware of potential complications to patient care.

Diagnosis via exclusion remains the primary method for the diagnosis of PCIS. The interval between the onset and diagnosis of PCIS can be several days to several months; moreover, the onset symptoms vary greatly, which makes the diagnosis challenging [1, 12]. Our review shows that the predominant symptoms in patients with PCIS include fever, chest pain, and dyspnea. Examinations show elevated ESR and CRP, pleural and pericardial effusion, and infiltration of the pulmonary parenchyma. Electrocardiograms rarely record findings of typical acute pericarditis. Factors that could increase susceptibility to PCIS include viral infection, degree of myocardial injury, and history of pericarditis [12]. In a few predisposed subjects, some rare and serious clinical manifestations may occur. These include massive pleural effusion with hypoalbuminemia and hyponatraemia, massive pulmonary infiltrates with hypoxemia, even peripheral hematoma of the ascending aorta, and acute cerebral infarction [3] (Table 1). After excluding the diagnosis of tumor, tuberculosis and connective tissue disease, it was considered that the mild increase in ESR is related to age. Consistent with most of the patients with PCIS, this patient also had elevated CRP, pleural and pericardial effusion, and infiltration of the pulmonary parenchyma [12].

**Table 1:** Clinical characteristics of post-cardiac injury syndrome reported in the literature

Study	Study design	Gender/age	Operation	Clinical symptoms	Examination	Treatment	PMID
**CIED implantation**							
Filbey 2020 [2]	Cohort	M(70.5%), 68.4(±13.3)	CIED implantation (incidence of 0.38%)	Dyspnea, chest pain, dry cough, and low-grade fever	ESR, leukocytes and CRP increases.	Pericardiocentesis, steroids or NSAID + steroids	32170340
Tadashi 2017 [5]	Case report	M,87	CRT implantation	low‐grade fever	Elevation of inflammatory marker	Colchicine	28588855
**PCI**							
Wang 2020 [3]	Case report	M,40	PCI	ARDS, vomiting, heavy sweating, and wet and cold limbs with irritability	ECG showed bow-back downward ST elevation in leads I, II, III, and V_4_-V_6_. ESR and CRP increases.	Prednisone 20 mg/d	32620081
Greener 2017 [6]	Case report	M,72	Chronic total occlusion-PCI	Severe pleuritic chest pain and a low-grade fever	ESR, leukocytes and CRP increased. Hemoglobin drop	High dose of aspirin and colchicine, clopidogrel.	28588855
**Transcatheter valve replacement/repair**							
Alachkar 2020 [4]	Case report	F,79	Transcatheter mitral valve repair	Fatigue, malaise, and non-specific epigastric pain	CRP increased and normal procalcitonin. CMR showed thickened pericardium with late gadolinium enhancement of both pericardium and pleura	High dose Aspirin (3g/day) and Colchicine (0.5 mg/day)	32974454
Llubani 2018 [7]	Case report	F,79	TAVI	Chest pain, dyspnea at rest, cough, and low-grade fever	Values of leucocytes and CRP were increased	Colchicine 0.5 mg, prednisone 20 mg, and ibuprofen 800 mg daily	31020183
**Catheter ablation**							
Davtyan 2022 [11]	Case report	F,66	ventricular arrhythmias	Dull oppressive non-radiating retrosternal chest pain	CMR showed local accumulation of pericardial fluid in front of the RVOT anterior wall, accompanied by adjacent oedema and pericardial contrast enhancement. ESR, leukocytes and CRP normal.	Diclofenac sodium 75 mg/daily	35028502
Wenzl 2021 [10]	Case report	F,62	Right septal ablation for AVNRT	Low-grade fever, chest pain on inspiration	Cardiac markers and leukocytosis increases. ECG showed diffuse saddle-shaped ST segment elevations.	Aspirin and low-dose colchicine	34953495
Li 2019 [8]	Case reports	M,82	Persistent AF	Poor appetite, fatigue and low-grade fever	ESR, CRP and NT-proBNP increases. There were marked hyponatremias (111 mmol/L, nor- mal value 137–145 mmol/L) and hypoalbuminemia (25 g/ L, normal value 35–50 g/L). Serious hypoxemia with arterial blood gas values as follows: pH, 7.54; PaO2, 71.25 mmHg; PaCO2, 38.7 mmHg.	Left-side thoracentesis, prednisone (10 mg, bid) and human serum albumin (10 g per day)	30322760
		M,78	Persistent AF	Cough, dyspnea and fever	CRP and NT-proBNP increases.	Prednisone (10 mg, bid)	
Han 2016 [9]	Case report	F,68	AF	Developed cough and mild chest discomfort	ESR, percentage of eosinophilic granulocytes and CRP increases.	Corticosteroid for half a month	30546651

*Abbreviations*: *M* Male, *F* Female, *AVNRT* Atrioventricular nodal reentrant tachycardia, *AF* Atrial fibrillation, *ARDS* Acute respiratory distress, *TAVI* Transcatheter aortic valve implantation, *CIED* Cardiac implantable electronic device, *CRT* Cardiac resynchronization therapy, *CMR* Cardiac magnetic resonance, *PCI* Percutaneous coronary intervention, *CRP* C-reactive protein, *NT-proBNP* N-Ter-minal Pro-B-Type Natriuretic Peptide, *ESR* Erythrocyte sedimentation rate

However, this patient presented with severe PAH, and the presence of concurrent severe TR is extremely rare, which made the diagnosis challenging. They must be differentiated from common diseases as well as in the following special cases: First of all, CTI ablation is a safe, effective, and well-established ablation procedure [13]. For patients undergoing CTI ablation, mechanical injury of the tricuspid valve is almost impossible. Currently, there are no reported cases in which CTI ablation has been associated with any mechanical damage to the tricuspid valve. And there are no single-sided valvular dysfunctions mentioned by ECHO. Tricuspid valve injury does not cause pulmonary hypertension. Furthermore, steroids might quickly treat PCIS but not the severe TR caused by the tricuspid valve mechanical lesion. Hence, mechanical harm brought on by CTI can be excluded. Secondly, stiff left atrial syndrome (SLAS) is a well-known complication that might follow extensive LA ablation [13, 14]. After ablation, SLAS developed with dyspnea, congestive heart failure, PAH, and diastolic dysfunction of the LA. This PCIS case needs to be differentiated from it because the symptoms and signs are confusing. There are major differences in this PCIS case from stiff left atrial syndrome: (1) PCIS has exhibited increased IL-6 and CRP, infiltration of the pulmonary parenchyma, pericardial effusion and pericardial thickening, but not in stiff left atrial syndrome; (2) In SLAS, the common ECG manifests no distinct P-wave or a reduction in the amplitude of the F-wave in AF or Atrial flutter. SLAS needs advanced echocardiographic parameters, including speckle tracking analysis and LA reservoir strain, for a differential diagnosis [14]. Last but not least, because of the patient’s dyspnea, elevated inflammatory markers, and lung exudation on CT, COVID-19 should be considered for the differential diagnosis at the time of the COVID-19 global pandemic. But the patient’s SARS-CoV-2 antigen was negative, and there was no history of exposure to the epidemic area. COVID-19 can therefore be disregarded.

Currently, most studies have reported that congenital and adaptive immune response inflammation phenotypes are the causes of PCIS [12, 15]. The exposure of endogenous antigens after heart injury leads to autoimmune and inflammatory reactions. The levels of anti-antibodies following cardiac damage may enhance and activate adaptive immunity and autoimmune T cells in PCIS patients [15]. In this patient, there were many ablation targets that released the cardiac autoantigen, which might combine with anti-antibodies in vivo to form an antigen-antibody complex and trigger an autoimmune reaction [12, 16]. This might be the mechanism of PCIS. Therefore, IL-6 would increase significantly due to the above factors. At the same time, IL-6 might be an important mediator in PAH [17, 18]. This might be a potential mechanism in this PCIS patient. The rare complications of this case may have been caused by local immune inflammation due to the deposition of an antigen-antibody complex in the pulmonary circulatory system, resulting in a surge of pulmonary artery pressure, a secondary increase in right ventricular volume load, and relative tricuspid insufficiency. Additionally, it was relieved after anti-immune treatment, so it was considered severe pulmonary hypertension and secondary severe tricuspid regurgitation, rather than organic tricuspid insufficiency.

Treatment of PCIS includes nonsteroidal anti-inflammatory drugs, colchicine, and steroids. If the above treatments are ineffective or contraindicated, azathioprine, immunoglobulin, and anakinra can be selected. It is rational to use steroids for patients who also need anticoagulant therapy [19]. Timely discovery and medical treatment of PCIS often result in the complete resolution of clinical symptoms, as well as the prevention of long-term complications.

The manifestations of PCIS vary, as this patient presented with severe pulmonary hypertension accompanied by severe tricuspid regurgitation. Due to the lack of diagnostic criteria, such patients are easily misdiagnosed, leading to a poor prognosis.

## Supplementary Information

**Additional file 1. **Representative images of parasternal long axis from echocardiography before pulmonary vein isolation (A) and during PCIS (B). No evidence of progressive aortic stenosis was found. (A) Aortic annulus diameter: 23mm. (B) Aortic annulus diameter: 24mm.

## Data Availability

Not applicable.

## Abbreviations

PCIS: Postcardiac injury syndrome
PAH: Pulmonary arterialhypertension
TR: Tricuspid regurgitation
RFCA: Radiofrequency catheter ablation
persAF: Persistent atrial fibrillation
CTA: Computed tomography angiography
ECHO: Echocardiography
LA: Left atrium
PVs: Pulmonary veins
CTI: Cavo-tricuspid isthmus
ECG: Electrocardiogram
SR: Showed sinus rhythm
ESR: Erythrocyte sedimentation rate
CRP: C-reactive protein concentration
IL-6: Interleukin-6
SLAS: Stiff left atrial syndrome
