# Supplementary figures and images for: Postcardiac injury syndrome caused by radiofrequency catheter ablation of persistent atrial fibrillation: severe pulmonary arterial hypertension with severe tricuspid regurgitation: a rare case report and literature review
Source: BMC Cardiovasc Disord. 2023 Apr 13;23:192. doi: 10.1186/s12872-023-03202-1 (PMC10100608; doi:10.1186/s12872-023-03202-1)

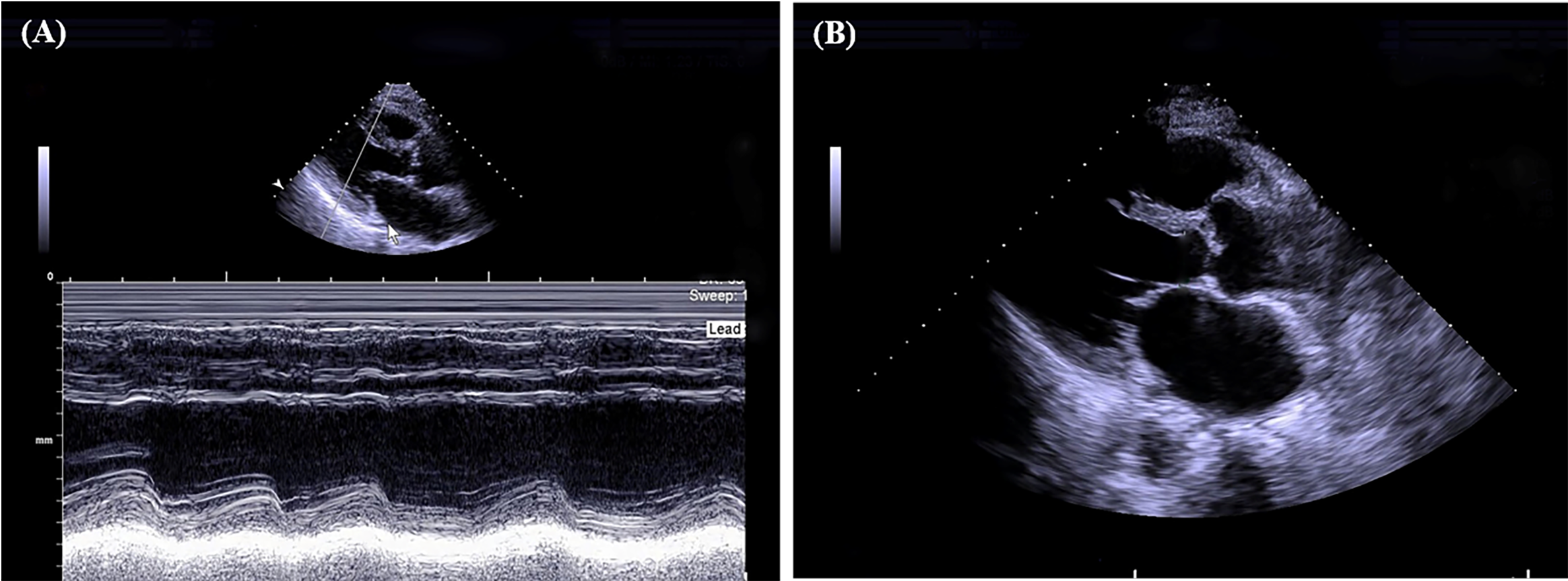

Supplement: Supplementary file 1 — Additional file 1. Representative images of parasternal long axis from echocardiography before pulmonary vein isolation (A) and during PCIS (B). No evidence of progressive aortic stenosis was found. (A) Aortic annulus diameter: 23mm. (B) Aortic annulus diameter: 24mm. [file 12872_2023_3202_MOESM1_ESM.tif]
